# Promotion and Prevention Focused Feeding Strategies: Exploring the Effects on Healthy and Unhealthy Child Eating

**DOI:** 10.1155/2015/306306

**Published:** 2015-08-25

**Authors:** Elisabeth L. Melbye, Håvard Hansen

**Affiliations:** UiS Business School, University of Stavanger, 4036 Stavanger, Norway

## Abstract

There is a general lack of research addressing the *motivations* behind parental use of various feeding practices. Therefore, the present work aims to extend the current literature on parent-child feeding interactions by integrating the traditional developmental psychological perspective on feeding practices with elements of Regulatory Focus Theory (RFT) derived from the field of motivational psychology. In this paper, we seek to explain associations between parental feeding practices and child (un)healthy eating behaviors by categorizing parental feeding practices into promotion and prevention focused strategies, thus exploring parent-child feeding interactions within the framework of RFT. Our analyses partly supported the idea that (1) child healthy eating is positively associated with feeding practices characterized as promotion focused, and (2) child unhealthy eating is negatively associated with feeding practices characterized as prevention focused. However, a general observation following from our results suggests that parents' major driving forces behind reducing children's consumption of unhealthy food items and increasing their consumption of healthy food items are strategies that motivate rather than restrict. In particular, parents' provision of a healthy home food environment seems to be essential for child eating.

## 1. Introduction

To both nutritionists and consumer researchers, it seems obvious that parents play an important role in child eating. They influence their children's diet and eating behaviors in many different ways, especially through their food-related parenting practices, or so-called feeding practices, which are specific techniques and behaviors used by parents to influence children's food intake [1, 2]. A number of studies provide evidence for a relationship between feeding practices, child eating, and child weight [3–8]. While parents' feeding practices have evolved from times when food scarcity was a major threat to children's growth and development, current food environments are characterized by ready availability of inexpensive, palatable foods, with high energy content but low nutrient density. Thus, we might say that feeding practices have developed from focusing on offering enough food to focusing on restriction of unhealthy foods and selection among the vast amount of food items available.

In the literature on public health and child nutrition, feeding practices have traditionally been categorized into different “feeding styles” corresponding with Baumrind's [9] taxonomy of parenting styles: authoritative, authoritarian, and permissive/neglectful. Parents with an authoritative feeding style encourage their children to eat healthy foods, but the children are also given some choices about eating options. In other words, parents determine which foods are offered and children determine which foods are eaten. Authoritarian feeding is characterized by parental control of child eating behaviors with little regard for the child's preferences and choices. This strictly regulatory style includes behaviors such as restricting certain foods (e.g., sweets and desserts) and forcing the child to eat other foods (e.g., vegetables). Parents with a permissive feeding style (also termed “nutritional neglect”) tend to allow the child to eat whatever he or she wants in whatever quantities wanted. Permissive feeding provides little or no structure and control, and the child's food choices are limited only by what is available [2].

This traditional developmental psychological perspective on parental feeding practices has been fruitful for our understanding of how the behavior of parents influences the eating behaviors of their children and also the effects this has on child food choice and weight. However, studying* what* parents do in relation to the food their children consume does not give a comprehensive picture of* why* they do what they do. While research on consumer psychology has focused on the underlying motives behind a large variety of human behaviors, there is still a shortage of research on parents' underlying motivations for applying the different feeding practices and styles [10]. Recently, though, a few studies have brought the motivational side to our attention, of which one is Carnell et al.'s [11] qualitative study among parents of 3–5-year-olds. Here, parents' feeding behaviors were found to be motivated most frequently by concerns for child health or by practical considerations (e.g., that child eating should fit into the family life and other requirements imposed on the parents' time). However, due to the exploratory nature of this work, no clear theoretical basis or conceptual model concerning parents' motivations for applying the different feeding practices was included. Another interesting study is Hingle et al.'s [10] research on parents' underlying motivations for using various vegetable parenting practices. Here, an adapted Model of Goal Directed Behavior (MGDB) [12] was used as the theoretical basis for qualitative interviews with parents of 3–5-year-old children. Subsequently, the results of this research were used to generate items and scales within a “Model of Goal Directed Vegetable Parenting Practices” to provide potential determinants of parental use of various vegetable feeding practices [13].

Based on the preceding paragraphs, we see a gap in the feeding literature concerning parents' motivations behind the application of various feeding practices. Thus, in the present study we turn to motivational psychology and Higgins' [14] Regulatory Focus Theory (RFT) to shed light on supplementary theoretical explanations for associations found in parent-child food-related interactions. RFT represents a highly influential paradigm in the growing research on self-regulation [15] and has been applied in a variety of studies on decision making [16], motivation [17], and consumer psychology [18]. RFT suggests that two different motivational systems drive people towards the attainment of desired outcomes. To achieve these outcomes, people may choose to either* approach* positive outcomes or* avoid* negative outcomes. The approach of positive outcomes is termed* promotion focus*, while the avoidance of negative outcomes is called* prevention focus*. According to the general idea of a “hedonic principle,” [19] people have a general tendency to approach pleasure (positive outcomes) and avoid pain (negative outcomes) [19–21]. It is reasonable to assume that this general tendency also applies to parenting practices, implying that parenting practices are generally driven by parents' desires for promoting positive outcomes and preventing negative outcomes for their children. Translated to the feeding domain, this may apply to parents' promotion of a healthy diet and prevention of an unhealthy diet for their children. According to Manian et al. [22], “there have been no studies specifically linking parenting behaviors with models of self-regulation” (p. 1622). Furthermore, Keller [23] considered the relationship between parenting styles and the specific self-regulatory orientations proposed in RFT “an important topic on the research agenda” (p. 357). Consequently, Keller assessed the relationship between parenting styles and self-regulatory orientations proposed in RFT in male university students. The assessment was based on students' self-reports on their own regulatory focus and recollections of their parents' child-rearing behaviors (reflecting retrospective ratings of parenting styles) and suggested that an authoritarian parenting style was associated with a chronic prevention focus and that an authoritative parenting style was associated with a chronic promotion focus. The permissive/neglectful parenting style, on the other hand, seemed to be of no critical importance with respect to the development of regulatory focus.

As far as we know, no studies have looked at parental feeding practices in relation to RFT. Thus, the objective of the present work is to build upon and extend the current literature on parent-child feeding interactions by integrating the traditional perspective on feeding practices with elements of RFT. In other words, we seek to explain associations between parental feeding practices and child (un)healthy eating behaviors by categorizing parental feeding practices into promotion and prevention focused strategies, thus exploring parent-child feeding interactions within the framework of RFT. Since we presume that feeding practices are generally driven by parents' desire of promoting positive outcomes (healthy eating) and preventing negative outcomes (unhealthy eating) for their children, our general assumption is that (1) feeding practices categorized as promotion focused are positively associated with healthy eating, and (2) feeding practices categorized as prevention focused are negatively associated with unhealthy eating. However, there are also reasons to believe that the associations between feeding practices and child eating are not as clear-cut as this, so our exploration will focus on both motivations (promotion/prevention) and both eating categories (healthy/unhealthy).

## 2. Materials and Methods

### 2.1. Participants and Procedures

To address the objectives of the present study, a cross-sectional survey directed towards 10–12-year-old children and their parents was performed. The rationale for focusing on 10–12-year-olds was twofold: firstly, children at this age are still highly influenced by their parents; secondly, they have made major cognitive advances compared to younger children, which facilitate their ability to report their behaviors. For practical reasons, a convenience sample was formed by recruiting participants through primary schools in two neighboring municipalities in southwest Norway. All primary schools in these municipalities were asked to participate in the study, and 18 out of 25 schools (72%) agreed. In total, 1466 grade 5 and 6 students and one of their parents were invited. First, parents' survey packages including information letters, consent forms, and self-administered questionnaires were distributed to the children at school with instructions to bring them home to be completed by one of their parents (the parent included was chosen by self-selection according to involvement in home food issues). Next, after receiving written consent from the parents, child questionnaires were distributed and completed by the students at school. Data collection took place from October to December 2009. The study was approved by the Norwegian Social Sciences Data Services (NSD), which is the Privacy Ombudsman for all the Norwegian universities, university colleges, and several hospitals and research institutes.

We received 963 completed parent questionnaires (66%). Response rate ranged from 44 to 93% among participating schools. Of the 963 parent respondents, 85% were mothers. The average age of the parents was 39.8 years, and 91% of the sample was of Norwegian or other Nordic origins. Out of 865 students having written consent from their parents to participate in the study, 796 (92%) completed the child questionnaire. Of the 796 child respondents, 51% were girls. Average age was 10.8 years. See Table 1 for more detailed sample characteristics.

### 2.2. Measures

Both parent and child draft questionnaires, which were largely based on items and scales from previous studies, were pretested before running the main survey. The drafts were tested through interviews with parents (*n* = 6) and students (*n* = 8) not included in the main survey to check if any questions, wordings, or scales were perceived as difficult to understand, easy to misunderstand, vague or ambiguous, strange, “stupid,” or irrelevant. Alternative wordings, scales, or ways of asking questions were discussed with them to enhance the understanding and relevance of the questionnaire for the target groups (Norwegian 10–12-year-olds and their parents). Feedback from parents and students was registered in a form developed for this purpose, and we continued to recruit pretest participants for interviews until no new feedback was given. Based on results from the pretest, the draft questionnaires were slightly modified to fit our populations of interest.

#### 2.2.1. Parent Questionnaire

The parent questionnaire included feeding scales from a Norwegian version of the Comprehensive Feeding Practices Questionnaire (CFPQ) [24, 25]. The CFPQ is a comprehensive feeding measure comprising 12 dimensions on parental feeding practices, of which 6 were included in the present study. Of the 6 feeding practices included, 3 were classified as promotion focused, targeting a desired outcome (healthy eating) for the child, and 3 were classified as prevention focused, steering away from an unwanted outcome (unhealthy eating) for the child. The promotion focused feeding practices measured were* encourage balance and variety* (parents promote well-balanced food intake, including the consumption of varied foods and healthy food choices),* healthy home food environment* (parents make healthy foods available in the home), and* teaching about nutrition* (parents use explicit didactic techniques to encourage the consumption of healthy foods). The prevention focused feeding practices measured were* monitoring* (parents keep track of their child's intake of less healthy foods),* restriction for health* (parents control the child's food intake with the purpose of limiting less healthy foods and sweets), and* restriction for weight* (parents control the child's food intake with the purpose of decreasing or maintaining the child's weight). A validation study by Melbye et al. [25] largely supports the validity of the CFPQ with parents of 10–12-year-olds in a Norwegian setting.

#### 2.2.2. Child Questionnaire

The child questionnaire included frequency questions adapted from the work of Andersen et al. [26]. The present study included two questions about healthy eating, represented by consumption of vegetables (How often do you eat vegetables for dinner? and How often do you eat other vegetables?), and one question about unhealthy eating, represented by the consumption of sugar sweetened beverages (SSB) (How often do you drink SSB like soda and lemonade?). The questions had 10 response categories (never = 1, less than once a week = 2, once a week = 3, twice a week = 4,…, six times a week = 8, every day = 9, and several times every day = 10). The children were asked to have their usual habits in mind when answering the questions. As suggested by Bere et al. [27], the 10 response categories were recoded to reflect vegetable and SSB consumption in times per week prior to data analyses (never = 0 times a week, less than once a week = 0.5 times a week, once a week = 1 time a week,…, every  day = 7 times a week, and several times every day = 10 times a week). Thus, all response categories had a common denominator (times a week), which improved the readability of the results, and increased comparability with studies using a similar consumption measure [26, 27].

#### 2.2.3. Data Analyses

The distribution of scores on each scaling variable was assessed by calculating mean, standard deviation, skewness, and kurtosis values. As suggested by Kline [28], we chose to apply cut-off values of 3.0 and 8.0 for skewness and kurtosis, respectively. Cronbach's alpha coefficients were computed to measure internal consistency of the scales. Bivariate correlation analyses were run to test for multicollinearity between independent variables. We applied a cut-off value of 0.80 or greater for multicollinearity, as suggested by Haerens et al. [29].

Only parent-child dyads with complete data sets for the associations tested were included in regression analyses (healthy eating/vegetable model: *n* = 671, unhealthy eating/SSB model: *n* = 697). To examine associations between parental feeding strategies and child consumption of vegetables and SSB, linear regression analyses were conducted with child self-reported vegetable and SSB consumption as dependent variables.

## 3. Results

Mean scores, standard deviations, skewness, kurtosis, and Cronbach's alphas for the study variables are presented in Table 2. Screening for skewness and kurtosis showed that all variables had values well within the range of chosen cut-offs. Cronbach's alphas ranged from 0.44 to 0.84. No multicollinearities were found between the independent variables. Correlations between study variables are presented in Table 3.

Results from regressions on healthy eating (i.e., vegetable consumption) and unhealthy eating (i.e., SSB consumption) are presented in Tables 4 and 5, respectively. Child healthy eating was positively associated with two out of three feeding practices characterized as promotion focused (i.e., strategies targeting a desired outcome):* encourage balance and variety* (*β* = 0.13, *P* < 0.01) and* healthy home food environment* (*β* = 0.15, *P* < 0.001). Moreover, two of the promotion focused practices were negatively related to unhealthy eating:* healthy home food environment* (*β* = −0.09, *P* < 0.05) and* teaching about nutrition* (*β* = −0.11, *P* < 0.01).

Child unhealthy eating was negatively associated with one of the three feeding practices characterized as prevention focused, namely,* monitoring* (*β* = −0.08, *P* < 0.05), while one of the prevention focused strategies (*restriction for health*) was also related to healthy eating (*β* = −0.14, *P* < 0.01). The results from regressions on child healthy eating (vegetable consumption) were previously published by Melbye et al. [30]. The results from regressions on child unhealthy eating (SSB consumption) are not previously published.

## 4. Discussion

The general lack of research addressing motivations behind parental use of various feeding practices was the impetus of the present work, where a traditional perspective on feeding practices obtained from the child feeding literature was integrated with a new, supplementary perspective obtained from motivational psychology. Our analyses partly supported the idea that (1) child healthy eating is positively associated with feeding practices characterized as promotion focused, and (2) child unhealthy eating is negatively associated with feeding practices characterized as prevention focused. However, not all our tested associations were significant, and the results also show that promotion focused strategies were related to* unhealthy* eating—in fact more closely related than prevention focused strategies. The succeeding paragraphs discuss these results in more detail.

The motivation behind the behavior parents portray in relation to their children's intake of various foods can have significant impact on a variety of issues, like present eating behaviors, future diet preferences, and general food habits. For example, research on RFT shows that our choice behavior as consumers varies with our motivational focus. A general observation following from our results suggests that parents' major driving forces behind reducing children's consumption of unhealthy food items and increasing their consumption of healthy food items are strategies that motivate rather than restrict. From a theoretical point of view, this corresponds with what consumer researchers call positive motivation [31], and it implies that both increased healthy eating and reduced unhealthy eating are driven mainly by the promotion focused strategies. From Table 4 we see that both the promotion focused strategies of encouraging balance and variety and providing a healthy home food environment positively affect healthy eating. Furthermore, the healthy home food environment also reduces consumption of unhealthy products, as does teaching about nutrition (see Table 5). Additionally, the prevention focused strategy monitoring is negatively associated with unhealthy eating, which seems logical as children have a less developed understanding of the negative effects of unhealthy eating than their parents and are thus less able to decide when enough is enough.

Although the results of our study were more mixed than expected, we nevertheless find reasons to suggest that child consumption of both healthy and unhealthy food items is primarily influenced by positive parental motivations expressed as promotion focused feeding strategies. In particular, the feeding variable* healthy home food environment* seems to be essential. The importance of a healthy home food environment is also discussed by Melbye et al. [32] who found a positive association between this variable and the frequency of shared family meals (which is positively associated with child healthy eating) and who suggested that parents providing a healthy home food environment will perhaps be more inclined to see the importance of sharing meals with their children. Correspondingly, we suggest that parents providing a healthy home food environment may be more liable to see the importance of encouraging their children to have a balanced and varied diet and to teach them about nutrition. Thus, one possible mechanism of the associations found may be that providing a healthy home food environment stimulates the application of other promotion focused feeding strategies which, in turn, leads to healthier child eating.

The negative association between restriction for health and child healthy eating is previously presented by Melbye et al. [30]. This result may at first glance seem unexpected, as one would intuitively assume a restrictive strategy to reduce unhealthy eating and have a* positive*, or no, effect on healthy eating. However, this is not what is found in the data. Melbye et al. find support for* mediation* of the association between restriction for health and child vegetable consumption by child self-efficacy. This finding is, in fact, not surprising, since parental restriction and control practices might have an unfavorable influence on child self-efficacy: the hypothesis is that restrictive parental practices might lead to less opportunity for the child to engage in activities that enhance his or her self-efficacy [33]. In this sample, parental restriction might reflect such a mechanism where children are not given sufficient opportunities to enhance their self-efficacy regarding food choice and eating behavior, including healthy eating, here resulting in a negative association between parental restriction for health and child vegetable consumption.

### 4.1. Strengths and Limitations

Among the strengths of this study is that it has reports from two different sources: parents and children. Thus, the “common methods problem” is reduced compared to situations where only one data source is available (e.g., parents reporting both feeding strategies and child eating). Another strength is its large sample size, which increases the validity of the results. However, some limitations should also be mentioned. First of all, this study does not include established motivation scales. We approach the motivation perspective by categorizing the measured feeding practices into promotion and prevention focused strategies based on the postulations presented by Higgins' RFT. Another limitation is the cross-sectional nature of the study, which makes causal inference impossible. Although our study indicates a model where parental feeding practices influence child eating (i.e., the “causal arrow” points from parent to child), we cannot exclude an alternative causal direction where parents are responding to their children's eating (i.e., the causal arrow points from child to parent). Nevertheless, the present work makes a contribution to the public health and child nutrition literature by giving attention to supplementary, and yet, underexplored theoretical explanations for associations found in parent-child food-related interactions.

### 4.2. Suggestions for Future Research

According to Pham and Chang [34], promotion focused consumers perform alternative search at a more global level than their prevention focused companions, and promotion focus is also associated with larger considerations sets than prevention focus. Related to child eating, this could imply that promotion focused feeding practices involve a larger variety of food items being considered, while a more narrow choice set is found with a prevention focus. Following this one step further, research on the cognitive mechanism on which RFT-effects are based suggests that there are also elaborational differences between promotion and prevention focused individuals. Zhu and Meyers-Levy [35] suggest that while a promotion focus is associated with relational elaboration, a prevention focused individual is more prone to perform item-specific elaboration. In relation to parental feeding practices, this could imply that promotion focused parents are more able to see the relationship between various parts of a diet and more globally judge the contribution each of these parts has on their goal (a healthy child diet). On the contrary, prevention focused parents could be more inclined to focus on item-specific food attributes without considering them in relation to other parts of the diet. Based on our empirical findings, we find a reason to believe that applying promotion focused feeding practices includes having a more relational view on eating behavior, thereby increasing healthy food consumption while simultaneously reducing unhealthy food consumption. Thus, feeding practices driven by an approach goal seem to accentuate healthy eating, but at the same time the attenuation of unhealthy eating appears to be a pleasant side effect. The prevention focused strategies seem to have limited effects on unhealthy eating, as it is only monitoring that is significantly related to child SSB consumption. This could correspond to an item-specific judgment where reducing unhealthy eating does not necessarily spill over to increases in healthy eating. However, our empirical data does not test, or support, such an explanation, but we will argue that future studies on parental feeding practices and child eating would produce fruitful insights on parent-child food-related interactions if such mechanisms and processes were further explored. The motivations for choosing different feeding practices and the kind of elaboration and choice set size and variation following from these would indeed be interesting avenues for future research.

## 5. Conclusions

Since parenting practices in general are assumed to be motivated by parents' desires for promoting positive outcomes and preventing negative outcomes for their children, it is reasonable to suggest that parents' focus on promotion versus prevention will influence the feeding practices they apply. Our results lend partial support to this assumption. Moreover, according to our findings, promotion focused strategies seem to be associated with increased consumption of healthy food items and decreased consumption of unhealthy ones. Thus, one implication from this study might be to encourage parents to use promotion focused strategies in food-related interactions with their children. However, further research on the drivers of various parental feeding practices is warranted, as this will increase our understanding of* why* and* when* different strategies are applied. Understanding such drivers, or underlying motivations, of parental food-related behaviors may offer new insights needed to develop more effective nutrition interventions tailored at parents and their children.

## Figures and Tables

**Table 1:** Sample characteristics.

Variable	Parents % or M (SD)	Students % or M (SD)
Age	39.8 (5.2)	10.8 (0.6)
Child gender		
Female		51%
Male		49%
Parental relation to child		
Mother	85%	
Father	12%	
Other caregivers (stepmother/-father)	1%	
Relation unknown	2%	
Parental ethnicity		
Norwegian/Nordic origin	91%	
Other (non-Nordic) origins	8%	
Origin unknown	1%	

**Table 2:** Means, standard deviations (SD), skewness, kurtosis, and Cronbach's alphas (*α*) for parental feeding strategies and child consumption of vegetables and SSB.

Variable/scale (number of items)	Mean	SD	Skewness	Kurtosis	*α*
*Parental feeding strategies *					
Encourage balance and variety (4)	4.47	0.51	−1.04	0.93	0.66
Home food environment (4)	3.92	0.68	−0.43	−0.28	0.57
Teaching about nutrition (3)	4.13	0.66	−0.67	−0.10	0.44
Monitoring (4)	4.05	0.56	−0.50	1.11	0.84
Restriction for health (4)	2.88	1.00	0.05	−0.78	0.73
Restriction for weight (8)	2.20	0.80	0.58	−0.08	0.83
Child consumption of vegetables (2)	5.48	2.22	−0.11	−0.80	0.52
Child consumption of SSB (1)	2.28	2.07	1.72	2.87	—

**Table 3:** Bivariate correlations between study variables.

	Enc. bal./var.	Home env.	Teach. nutr.	Monitoring	Rest. health	Rest. weight	Veg. cons.	SSB cons.
Enc. bal./var.	1							
Home env.	0.26^**^	1						
Teach. nutr.	0.52^**^	0.34^**^	1					
Monitoring	0.20^**^	0.16^**^	0.13^**^	1				
Rest. health	0.08^*^	−0.06	0.02	−0.04	1			
Rest. weight	0.09^*^	0.05	0.11^**^	0.01	0.56^**^	1		
Veg. cons.	0.18^**^	0.20^**^	0.15^**^	0.08^*^	−0.12^**^	−0.02	1	
SSB cons.	−0.08^*^	−0.13^**^	−0.14^**^	−0.11^**^	−0.01	0.00	−0.17^**^	1

^*^ *P* < .05; ^**^ *P* < .01.

**Table 4:** Results from regression analyses testing associations between parental feeding strategies and child vegetable consumption.

	Child consumption of vegetables (*β*)
*Promotion focused feeding strategies *	
Encourage balance and variety	0.13^*^
Home food environment	0.15^*^
Teaching about nutrition	0.04
*Prevention focused feeding strategies *	
Monitoring	0.01
Restriction for health	−0.14^*^
Restriction for weight	0.03

^*^ *P* < .01.

**Table 5:** Results from regression analyses testing associations between parental feeding strategies and child SSB consumption.

	Child consumption of SSB (*β*)
*Promotion focused feeding strategies *	
Encourage balance and variety	0.02
Home food environment	−0.09^*^
Teaching about nutrition	−0.11^*^
*Prevention focused feeding strategies *	
Monitoring	−0.08^*^
Restriction for health	−0.05
Restriction for weight	0.05

^*^ *P* < .05.
